# A Novel Soft Contact Piezo-Controlled Liquid Cell for Probing Polymer Films under Confinement using Synchrotron FTIR Microspectroscopy

**DOI:** 10.1038/s41598-018-34673-4

**Published:** 2018-12-13

**Authors:** Natalie L. Benbow, Jessie L. Webber, Piotr Pawliszak, Damien A. Sebben, Tracey T. M. Ho, Jitraporn Vongsvivut, Mark J. Tobin, Marta Krasowska, David A. Beattie

**Affiliations:** 1 Future Industries Institute, University of South Australia, Mawson Lakes, South Australia 5095 Australia; 2 School of Information Technology and Mathematical Sciences, University of South Australia, Mawson Lakes, South Australia 5095 Australia; 3 Infrared Microspectroscopy (IRM) Beamline, Australian Synchrotron, Clayton, Victoria 3168 Australia

## Abstract

Soft polymer films, such as polyelectrolyte multilayers (PEMs), are useful coatings in materials science. The properties of PEMs often rely on the degree of hydration, and therefore the study of these films in a hydrated state is critical to allow links to be drawn between their characteristics and performance in a particular application. In this work, we detail the development of a novel soft contact cell for studying hydrated PEMs (poly(sodium 4-styrenesulfonate)/poly(allylamine hydrochloride)) using FTIR microspectroscopy. FTIR spectroscopy can interrogate the nature of the polymer film and the hydration water contained therein. In addition to reporting spectra obtained for hydrated films confined at the solid-solid interface, we also report traditional ATR FTIR spectra of the multilayer. The spectra (microspectroscopy and ATR FTIR) reveal that the PEM film build-up proceeds as expected based on the layer-by-layer assembly methodology, with increasing signals from the polymer FTIR peaks with increasing bilayer number. In addition, the spectra obtained using the soft contact cell indicate that the PEM film hydration water has an environment/degree of hydrogen bonding that is affected by the chemistry of the multilayer polymers, based on differences in the spectra obtained for the hydration water within the film compared to that of bulk electrolyte.

## Introduction

Water is known as the universal solvent covering 71% of the earth’s surface^1^, and plays a necessary role in many chemical and biological systems. The structure and function of many soft matter systems is dependent on their interaction with water to maintain their desired properties and functionalities. Some of key examples include, proteins and enzymes within the body^2^, *in vitro* cell culture^3,4^ and biofilm formation^5^, hydrated thin films such as polyelectrolyte multilayers^6^, hydrogels^7–9^ and polymer brushes^10–12^, as well as aqueous catalysis^13,^ and water treatment membranes^14^. Therefore, the ability to study these soft materials in an aqueous environment is the critical factor to gain a better understanding of how such materials function.

Polyelectrolyte multilayers (PEMs) are a versatile and useful soft matter system, whose properties, including film thickness, density, surface charge and hydration content, can be controlled. There are a number of experimental techniques commonly used for studying the properties of hydrated PEMs in an aqueous environment, *e*.*g*. QCM-D^15–21^, AFM^22,23^, SPR^17,18^, ellipsometry^24^, combined QCM-D/ellipsometry^25^, NMR^26^, neutron reflectometry^27–29^ and ATR FTIR^6,16^. Of these techniques, only the last two are able to provide structural information of a PEM (through modelling of reflectivity curves in the case of the neutron reflectivity, and through analysis of spectral profiles in the case of ATR FTIR).

Although potentially very informative, ATR FTIR has a major limitation in studying hydrated PEMs, as the technique probes not only the thin film/soft matter region, but also the bulk solution above it (due to the penetration depth of the evanescent wave). It is possible to overcome this limitation by: (*i*) altering the methodology to probe a smaller sampling volume from the solid interface^6^, or (*ii*) by confining the film between two solid interfaces^19,21^. The former approach does not allow for the interrogation of very thin layers. Confinement between two solid surfaces enables the analysis of thin film properties without interference from bulk solutions adjacent to the film, and is the focus of this work.

While studies using physical confinement of polyelectrolyte multilayers are scarce, this particular approach has been used by Abbott^27^ and De Vos^28^, who used neutron reflectometry to assess hydration of odd-even terminated PEMs formed from synthetic polyelectrolytes. The drawback of using neutron reflectometry is the necessity to use a deuterated polyelectrolyte to provide the necessary contrast within the multilayer. A different approach was developed by Beattie *et al*.^19^, which eliminated the need for deuteration, thus expanding the capability of the technique for analysis of other systems where deuteration is not possible, such as polysaccharide PEM films. Beattie *et al*.^19^ used synchrotron Fourier-transform infrared (FTIR) microspectroscopy along with a custom built solid−solid contact cell to probe the structure of boundary lubricant layers confined between two solid surfaces. This method, however, lacked the precise control of pressure applied during the contact formation and was restricted to soft/flexible solid surfaces (*i*.*e*. mica, which is not ideal for FTIR studies due to its anisotropic optical properties). Ho *et al*.^21^ modified the approach to suit different solid surfaces, and used it to determine hydration and the hydrogen bonding environment of the water within PEMs formed by biopolymers. The results demonstrated that FTIR spectroscopy possessed a unique capability to probe the polymer characteristics and simultaneously monitor changes in hydration water within the PEMs^6,19,21^.

This work describes a further advancement in using synchrotron FTIR microspectroscopy to study confined polymer films in a hydrated environment, using a novel soft contact liquid cell. The liquid cell works by using a ZnSe hemisphere to form a controlled solid-solid interface with an underlying substrate. The substrate is brought into contact with the crystal using a unique combination of piezo-controlled linear translation stages in both *xy*- and *z*- directions. The minimum step interval of the piezo drive can be set as small as 50 nm, making it ideal for analysis of delicate and fragile materials. In addition, the geometry of the ATR crystal (curved under surface for contact) ensures that the application of pressure (of practical/relevant magnitude) is gentle and non-destructive. Such a unique design allows *in situ* monitoring of the hydration of the confined film under different (and controlled) degrees of compression.

A polyelectrolyte multilayer made of poly(sodium 4-styrenesulfonate) (PSS) and poly(allylamine hydrochloride) (PAH), was used in this study to demonstrate the application of this custom liquid cell specifically for probing the hydration of soft matter/thin films under controlled confinement. The PSS/PAH system was selected due to its simplicity^6^. Synthetic polyelectrolytes tend to have narrower polydispersity, and better defined chemical structures than naturally occurring polyelectrolytes. In addition, the build-up of these two synthetic polyelectrolytes tends to be linear, and their hydration has been thoroughly studied using various approaches^30^. Importantly, these two polyelectrolytes do not contain any hydroxyl (-O-H) groups in their structures, preventing interference in the band shape of the O-H stretching mode of water in the observed IR spectra. The synchrotron FTIR microspectroscopy data is complemented by lab-based ATR FTIR spectroscopy and streaming potential measurements, performed to confirm the nature of the multilayer build-up.

## Results and Discussion

### Multilayer Build-up

#### Zeta potential

The buildup of a subsequent layers of PSS/PAH film was confirmed in streaming potential experiments, where the determined zeta potential had a negative value for a multilayer terminated with polyanion, and a positive value (for a multilayer terminated with polycation. The zeta potential data as a function of the PEM terminating layer is presented in Supplementary Figure S1 along with a full description of the experimental protocol and detailed description of the data.

#### ATR-FTIR

**Figure 1:**
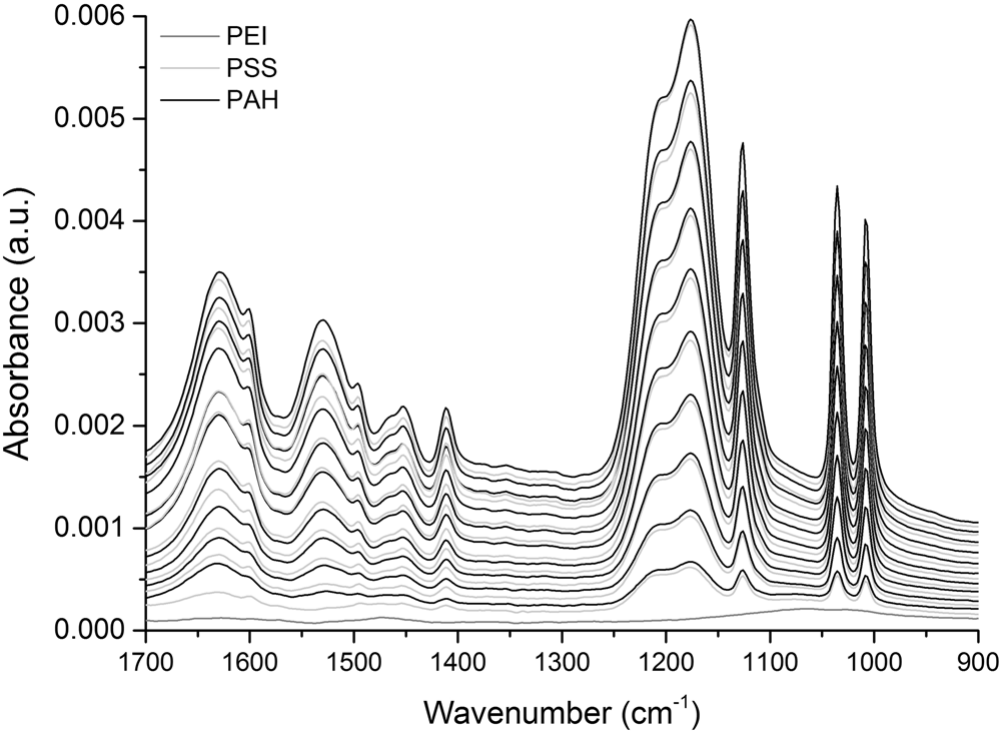
*In situ* ATR-FTIR spectra of the build-up of a 10 bilayer PSS/PAH multilayer, showing the region that contains the characteristic peaks for these polymers. Note: significant increases in intensity of the characteristic peak(s) of each polymer after its adsorption were observed. The dark grey trace represent the anchoring PEI layer, while the light grey and black traces represent PSS and PAH, respectively.

**Figure 2:**
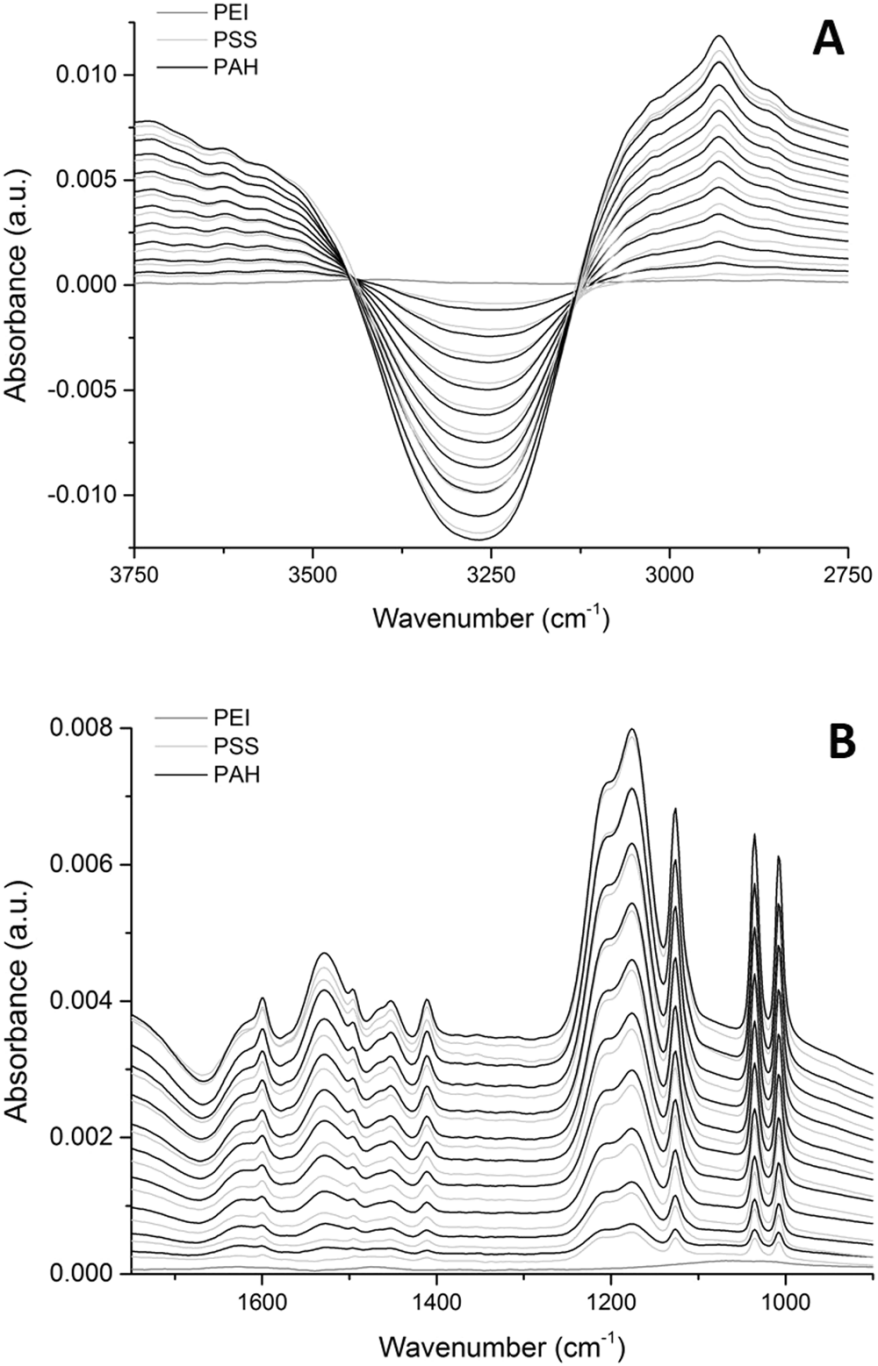
*In situ* ATR-FTIR spectra of the build-up of a 10 bilayer PSS/PAH multilayer where the background electrolyte spectrum was subtracted by a factor of 1. Panel (A) shows the O-H stretching mode region, whilst (**B**) displays the O-H bending mode and the region containing the characteristic peaks for these polymers. The dark grey trace represent the anchoring PEI layer, while the light grey and black traces represent PSS and PAH, respectively.

The ATR-FTIR spectra of a 10 bilayer PSS/PAH multilayer are presented in Figs 1 and 2. The anchoring PEI layer is represented by a dark grey line, while the successive PSS and PAH layers are shown in light grey and black, respectively. The fingerprint region within the spectral range of 1700–900 cm^−1^, is the focus of the multilayer build-up spectra, as presented in Fig. 1. This region contains the majority of the characteristic peaks of the two polymers in the multilayer (see Table 1). The multilayer build-up spectra were produced by manually subtracting the background electrolyte spectrum (collected prior to each experiment) from each spectrum (taken after a polyelectrolyte/rinse step) to remove the contributions from water in the O-H bending region. The peaks seen in the multilayer spectra are assigned based on published literature (see references in Table 1). PSS has a spectrum with prominent features, that consist of three sharp peaks (1008, 1036, 1126 cm^−1^) and some overlapping peaks (1180 and 1208 cm^−1^), all of which are assigned to S=O stretching modes. The characteristic peaks of PAH are less prominent, but the largest peaks are attributable to amide I (1626 cm^−1^), amide II (1527 cm^−1^), and C-H bending mode (1465 cm^−1^). Overall, the peak assignments of the multilayer spectra are directly comparable to the solution reference ATR-FTIR spectra, with a few exceptions including the shift of S=O stretching mode from 1180 cm^−1^ to 1175 cm^−1^.

**Table 1:** Peak maximum positions and assignments for ATR-FTIR spectrum of the individual polyelectrolytes (PSS and PAH), and the 10 bilayer polyelectrolyte multilayer.

Peak Assignment	PSS	PAH
alkyl C-H stretching	2931	
amide I		1629
aromatic -C=C- stretching	1601	
amide II		1530
aromatic -C=C- stretching	1495	
C-H bending		1465
aromatic -C=C- stretching	1453	
aromatic -C=C- stretching	1411	
-S=O anti-symmetric stretching overlapping with aromatic -C=C- bending	1208	
-SO_3_^−^ anti-symmetric stretching	1175	
-C-H aromatic	1126	
-SO_3_^−^ symmetric stretching	1036	
-C-H aromatic	1008	
=C-H deformation	834	
=C-H deformation	774	

References used for assignments are: for PAH; Li^50^, Cornejo^51^,and PSS; Świderski^52^, Yang^53^ and Zundel^54^; for both PAH and PSS; Coates^55^, and Stuart^56^.

The polyelectrolyte multilayer build-up is presented again in Fig. 2, although here the data is presented differently. The spectrum of each polyelectrolyte/rinse step has the background electrolyte subtracted in a 1:1 ratio. Panel (A) shows the O-H stretching mode region and panel (B) shows the fingerprint region, which also contains the O-H bending mode at (~1630 cm^−1^). Panel (A) shows a consistent decrease in the O-H stretching band as each polyelectrolyte layer is added. The O-H stretching band ‘dip’ increases in magnitude (moves further away from 0.000 absorbance) with increasing bilayer number as the polyelectrolyte multilayer displaces the water (background electrolyte) from within the region probed by the evanescent wave. Panel (B) reflects this with the O-H bending mode proportionally becoming more negative as well. This does not give information about the structure of water within the multilayer, but does show that the multilayer contains less water than bulk electrolyte (synthetic systems contain 20–75% hydration^31^). The water content of PSS/PAH films are not greatly affected by the salt concentration of background electrolytes during build-up^30^. It has been previously reported that PSS terminating multilayers have 28% hydration water, and PAH terminating multilayers contain 22% water^28^.

**Figure 3:**
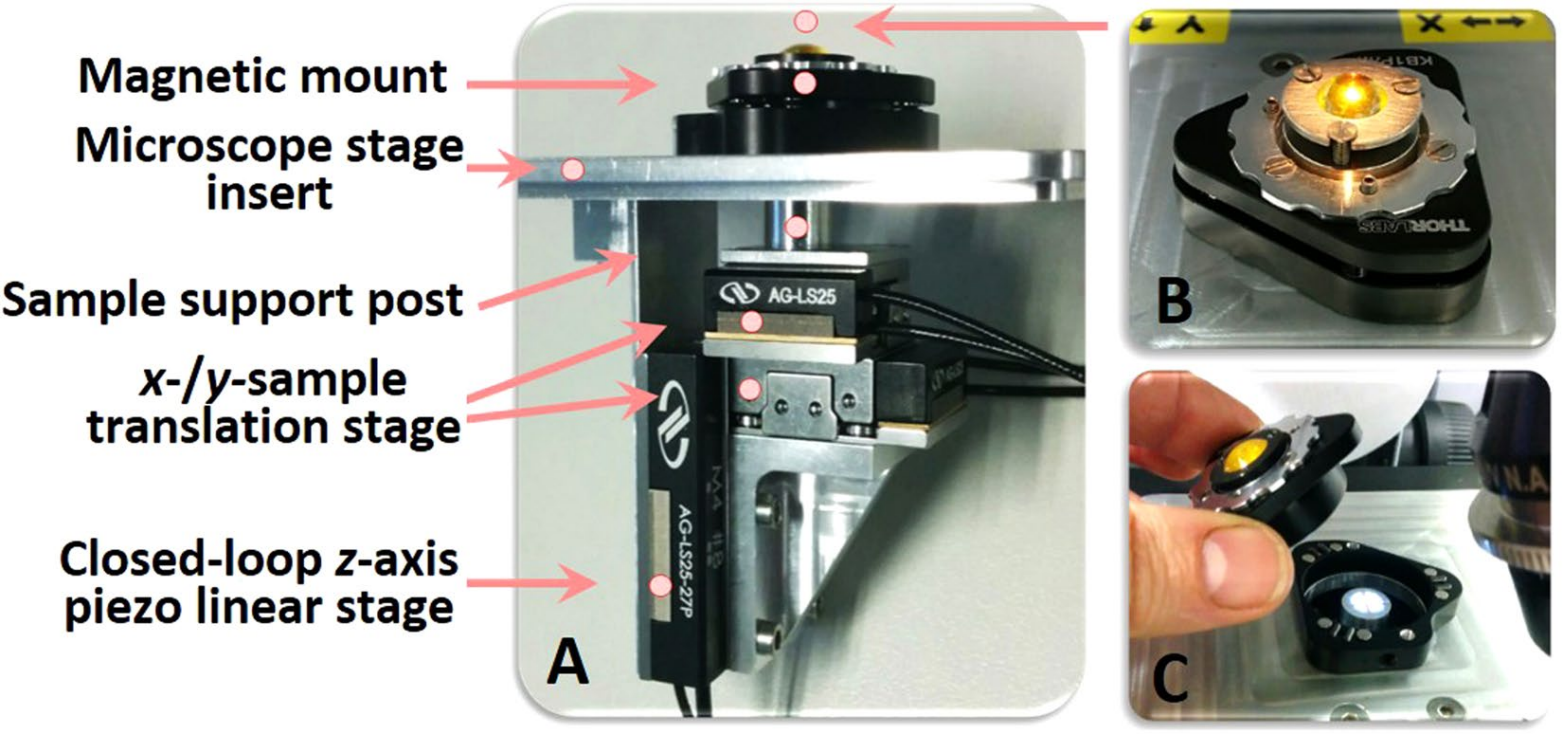
Photographs of the custom-built piezo-controlled liquid cell developed specifically for probing hydration in the PEM films. (**A**) The device consists of a unique combination of piezo-controlled linear translation stages used in both *xy*- and *z*- directions, to achieve a gentle and precise positioning of the film sample mounted on the support post, with the minimum step interval of the piezo drive set to be 50 nm. (**B**) The device uses a custom-designed ZnSe hemisphere having radius of curvature (ROC_*top*_) of 5 mm with a curved bottom surface (ROC_*bottom*_ = 20 mm), as an ATR crystal, to create Newton’s ring appearance indicative of the first contact between the PEM film and the ATR sensing surface. (**C**) A magnetic mount was the mechanism of choice used to hold the ZnSe hemisphere crystal, to achieve precise positioning of the crystal when changing the sample.

From the spectra in Fig. 3, it is clear that using a laboratory-based ATR-FTIR instrument with a single reflection ZnSe ATR element does not allow direct measurement of water trapped within a film because the evanescent wave can penetrate into the bulk electrolyte. Using this method, spectral manipulations must be used to visualize the polyelectrolyte peaks, but doing so distorts the O-H stretching (see Fig. 3A) and O-H bending (see Fig. 3B at ~1630 cm^−1^) modes of water within the multilayer. It is possible to circumvent this issue by using an alternate internal reflection element such as germanium, which has a higher refractive index (*n*(Ge) = 4 compared to *n*(ZnSe) = 2.4), resulting in a shallower evanescent wave penetration depth. This was done by Schlenoff *et al*.^6^, who investigated the internal hydration of synthetic polyelectrolyte multilayers. This method offers an easy way to probe the internal hydration of the multilayer but it does not allow the higher layers of the multilayer (that are outside the range of the evanescent wave penetration depth) to be analysed or, the analysis of very thin films (where the bulk water is within the range of the evanescent wave penetration depth).

### Synchrotron FTIR Microspectroscopy

#### Soft Contact Cell Design

Due to the novelty of the design, details on the new soft contact cell are provided here. A schematic of the cell is presented in Fig. 3. The samples are glued onto a 6-mm-diameter round magnetic steel disc and then placed onto the cylindrical magnetic sample holder on the support post (Fig. 3). The movement of the sample holder in *z*-direction is controlled by a closed-loop *z*-axis piezo linear stage, where the step interval was set to the minimum increment of 50 nm. This allows for precise control of the position of the sample and, hence, the compression of the films. An o-ring around the sample translation piston ensured the sealing of the liquid cell. The top part of the liquid cell device consists of a custom-designed ZnSe hemisphere (Crystran, UK) with two curved surfaces, held in a magnetic mount. The top curved surface of the ZnSe hemisphere with a radius of curvature (ROC_*top*_) of 5 mm, is the entry and exit surface for the IR beam. The bottom curved surface with a ROC_*bottom*_ of 20 mm, allows the formation of a contact spot of a defined area (radius approximately 150 μm) between the bottom sensing surface of the ZnSe hemisphere and the sample. The ZnSe hemisphere is mounted in a stainless steel frame with two o-rings: (*i*) one on the top of the crystal and being held in place by a plastic element mounted with three small screws to ensure a proper alignment of the optical element and, (*ii*) the one beneath the crystal to guarantee a leak-proof operation of the cell. The stainless steel frame supporting the adjustable ZnSe hemisphere was mounted directly above the sample translation piston using a 3-point magnetic optical mount (Thorlabs, Newton NJ, USA), which could be removed for sample loading, and replaced with a positional accuracy better than 10 μm. Full details of the specific sample preparation protocol and spectral acquisition for the PEM films are given in the Methods section.

It should be emphasised that the contact region is made between one curved and one flat surface, therefore the pressure (and thereby the hydration) across the contact varied across the contact spot. The maximum pressure is exerted at the center of the contact (where the hemisphere was closest to the gold substrate), whilst outside the contact region the hemisphere may not be in contact with the multilayer at all. The average pressure within the contact region can be calculated from the maximum pressure^19,21^. In principle, the sampling area for synchrotron FTIR microspectroscopy measurement is estimated to be within 3–8 μm in diameter, so measurements can be taken at very specific points across the contact in the *xy* plane, while the piezo-stage gives a fine control in the *z*-direction. It should be noted that precise alignment of the ZnSe hemisphere and the control of the sample position in the *z*-direction was not possible in previous experiments, and therefore this work represents a significant advancement in our methodology^19,21^.

One other point needs to be made concerning the acquisition of spectra of thin samples confined between the ZnSe hemisphere and the underlying substrate on which the sample is formed. Gold was used as the surface onto which the PEM films were formed in this work. The contact between the ZnSe hemisphere and the underlying gold will not give rise to the establishment of an evanescent wave at the surface of the ZnSe hemisphere. The optical interface is more akin to that seen in a surface plasmon resonance (SPR) instrument^3,4,32,33^, but in this case with the sample of interest sandwiched between the dielectric material (ZnSe) and the gold, rather than attached to the outer surface of the gold (which is the configuration for studying thin films with SPR). Therefore, in our system, the IR spectra can be viewed essentially as a double pass transmission experiment, with the IR light reaching the detector having passed through the sample, reflecting off the gold, and then passing through the sample again on the way back to the detector. This configuration has provided a far better signal-to-noise than previous iterations of this cell^21^, where a mica substrate was used as the support for the multilayer.

#### Multilayer Under Confinement

**Figure 4:**
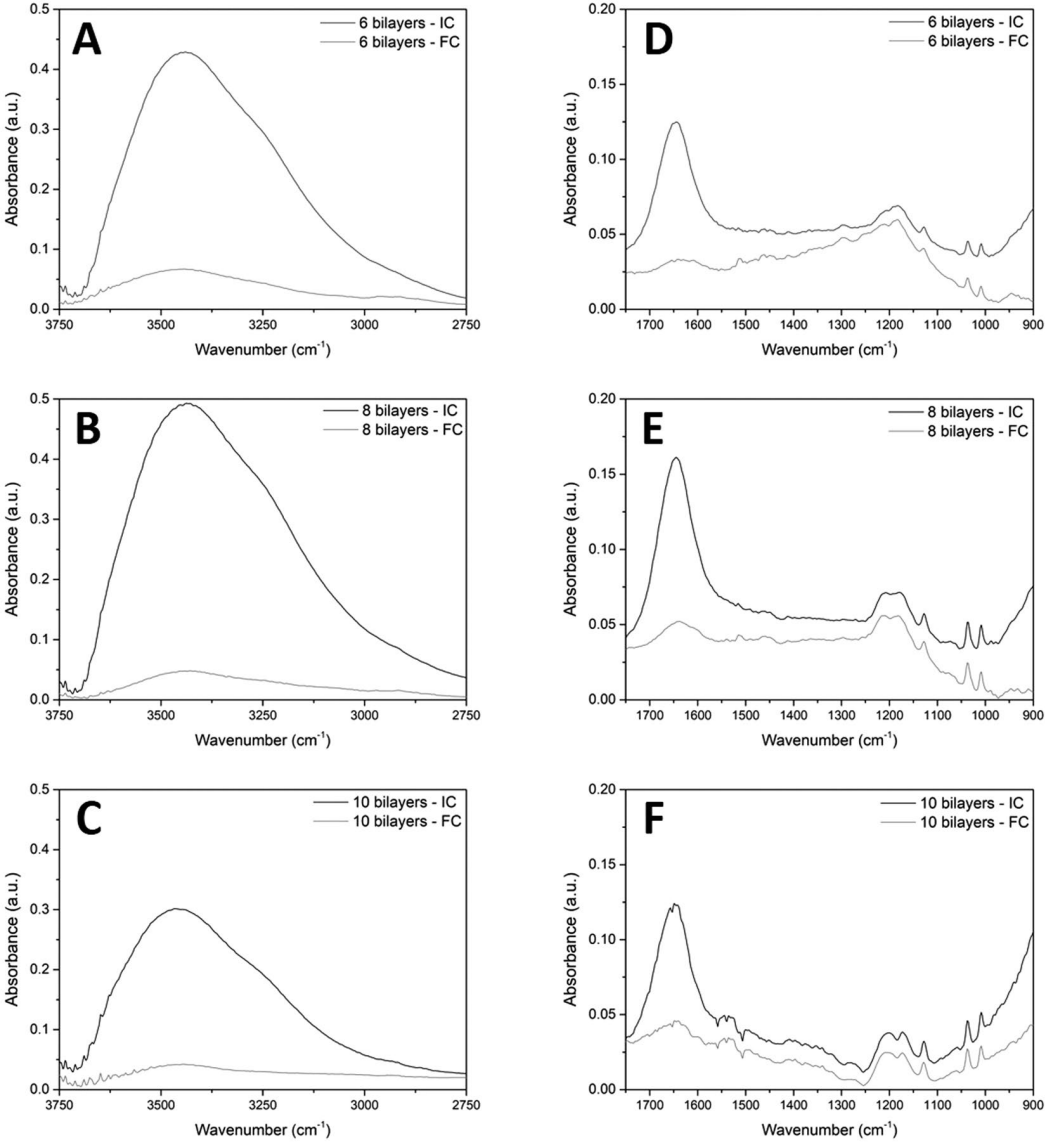
Synchrotron FTIR spectra of initial contact (IC) and the final compression (FC) for the O-H stretching region of (**A**) 6 bilayer, (**B**) 8 bilayer and (**C**) 10 bilayer multilayers. And, the corresponding fingerprint region containing the characteristic peaks shown in panels (**D**) 6 bilayer, (**E**) 8 bilayer and (**F**) 10 bilayer multilayers.

The spectra collected using the soft contact liquid cell and synchrotron FTIR are presented in Fig. 4, which is vertically divided into panels A, B, and C, showing the O-H stretching mode of bilayer numbers 6, 8 and 10 (PAH terminating), respectively. And panels (D, E and F) show the corresponding fingerprint region. The spectra were taken after the corresponding polyelectrolyte/rinse step, representative spectra are presented (see Supplementary Figure S2 for the full dataset). The spectra corresponding to the initial contact of the crystal with the multilayer and the final compression of the multilayer are presented in Fig. 4. In the case of Fig. 4, the only spectral processing was atmospheric water vapour and baseline correction.

The fingerprint region (Fig. 4 panels D, E and F) shows the characteristic peaks of the polyelectrolytes and the O-H bending mode. The polyelectrolyte peaks can be compared to the solution spectra collected using the lab-based ATR-FTIR. In the case of PSS, it is easy to identify the peaks assigned to S=O stretching vibrations at 1205, 1172, and 1037 cm^−1^, which correspond to the peak assignments given in Table 1. The amide I/II bands of PAH are not as clear in these spectra, compared to the spectra in Figs 2 and 3.

Two important points are revealed by considering the intensity of the multilayer peaks. Firstly, as the bilayer numbers increase from 6 bilayers, panel (D), to 10 bilayers, panel (F), an increase in absorbance of the S=O stretching modes can be seen as would be expected from multilayer build-up. Secondly, when compressed, the polyelectrolyte peaks do not change in size. This indicates that the crystal is in contact with the multilayer at initial contact, and when the film is compressed the multilayer remains intact. This also confirms that the signals from water (O-H stretching and bending modes) are from water trapped within the multilayer itself, not from bulk electrolyte between the crystal and the multilayer.

The O-H stretching regions of the multilayer spectra are shown in Fig. 4, panels A, B and C. Since there is no spectral processing (except for water vapour correction), the broad band between 3000–3800 cm^−1^ represents the O-H stretching mode of water within the PEM, and can be used to discern information about the water environment within the multilayer system. The O-H stretching mode of water can be deconvoluted into components that indicate the degree of hydrogen bonding of water, or the overall peak shape can be used to indicate the degree of hydrogen bonding of water within a specific environment, *i*.*e*. trapped within a polyelectrolyte multilayer. However, since there is much debate on the appropriateness of peak deconvolution of the O-H stretching region^21,34–41^, we restrict ourselves here to discussion on the general shape and peak maximum position of the overall O-H stretching mode.

As expected, the O-H stretching mode decreased as each sample was compressed, indicating that water was gradually removed from the multilayer (see Supplementary Figure S2 for the sequential compression of each multilayer, in 50 nm steps on the piezo drive). This is also reflected in the O-H bending mode seen in panels D, E and F of Fig. 4. It would be expected that the O-H stretching band of the initial contact would increase with increasing bilayer number as a thicker multilayer would hold more water than a thinner multilayer, however, this is not the case. The O-H stretching mode of bilayer 8 is larger than that of bilayer 6, but in the case of the 10 bilayer system, the O-H stretching mode has lower absorbance than either the 6 or 8 bilayer systems.

It is possible that the 8 bilayer system has properties that cause it to be more hydrated than the 10 bilayer system but it may also be due to the experimental technique itself. Due to limitations associated with the surface quality of the hemispheres and gold substrates used, and small variations in the positioning of the hemisphere, spectra could not always collected in the same place (*i*.*e*. ideally in the very centre) within the contact, due to scratches or unevenness of the gold substrate. This means that there may have been some variance in the pressure at the measurement spot used for each initial contact spectrum. The average pressure experienced by the film at initial contact was calculated to be approx. 160 MPa, whilst the maximum pressure in the centre would be 240 MPa (based on Hertzian contact theory^19^ using the radius of the contact, the radius of curvature of the hemisphere, the Young’s modulus and Poisson ratio of the ZnSe^19^ and of the Si wafer substrate^42^).

The final compression spectra in Fig. 4 (panels A, B, and C) represent only the water trapped within the multilayer, which was expected to increase with bilayer number. However, the data does not show an increase in the O-H stretching mode. Instead, as bilayer number increases the O-H stretching mode remains almost constant. Finally, when the shape of the O-H stretching band of the initial contact is compared to the final compression of each system, it can be noted that the overall band shape of the water within the multilayer does not change from initial contact through to the final compression, which shows only tightly bound/trapped water. This is in agreement with Schlenoff ^6^, who used FTIR spectroscopy to observe the O-H stretching mode lineshape of water within synthetic polyelectrolyte multilayers and bulk water. He proposed that since the water seemed to maintain its degree of hydrogen bonding whether in a multilayer or not, it was suggested that water molecules ‘cluster around the ionic functionality’.

#### Removed water versus bulk water comparison

**Figure 5:**
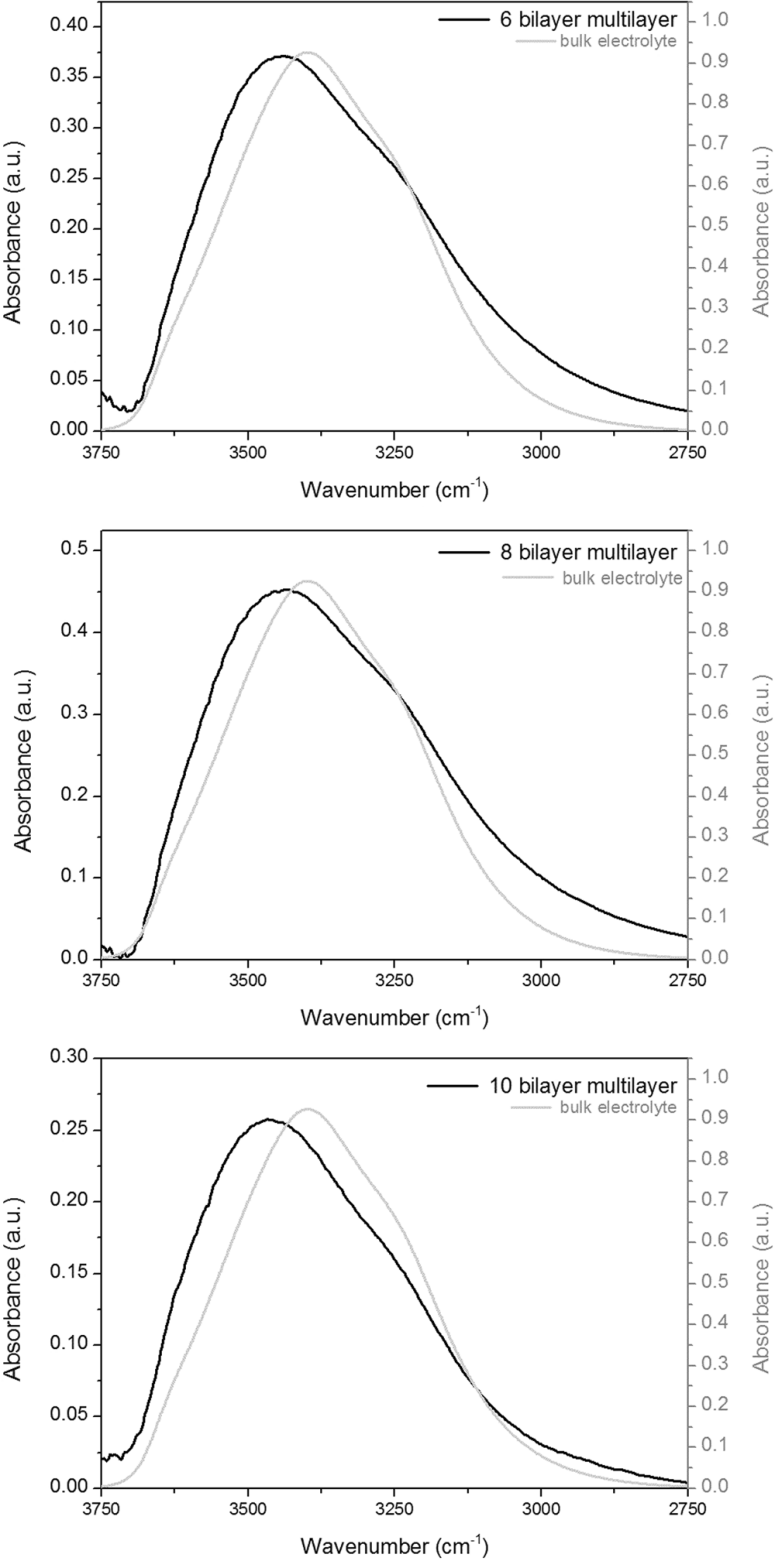
The light grey trace in each panel represents an ATR-FTIR spectrum of the bulk KCl electrolyte that was corrected for anomalous dispersion, whilst the black trace shows the ‘removed’ water which was produced by subtracting the final compression spectra from the initial contact spectra in a 1:1 ratio. Panel (A) shows the 6 bilayer system, (**B**) shows the 8 bilayer system, and (**C**) shows the 10 bilayer system. Note: different scales are used for each trace to best show the differences in peak shape between bulk electrolyte and water removed from the multilayer.

The spectra of the residual water contained within the compressed multilayers indicate that very little water remains once the multilayer experiences high pressures. The ability to acquire spectra of hydrated films upon initial contact, and after significant application of pressure, allows us to perform a simple spectral subtraction to see the characteristic profile of the water that has been removed during the sequential application of increasing pressure. The synchrotron FTIR spectra of the ‘removed water’ from each multilayer sample (6, 8, and 10 bilayers) can be seen in Fig. 5.

The profiles of the O-H stretching bands have a maximum at approx. 3465 cm^−1^, consistent for all multilayer samples. We have plotted in Fig. 5 the spectrum of bulk electrolyte obtained using a normal lab-based ATR FTIR experiment, using the same crystal as for the multilayer build-up spectra shown in earlier figures. To ensure that the comparison is meaningful, the ATR-FTIR spectrum of bulk electrolyte was corrected using an anomalous dispersion correction (using OMNIC software suite v8.2.0.387), which is necessary when studying broad strong absorbances with ATR-FTIR. As mentioned in the methodology section, the spectra acquired of the confined multilayers within the solid-solid contact (ZnSe-gold) are not true ATR spectra, and are essentially transmission spectra. As such, they do not require anomalous dispersion correction. It can be seen that the peak maximum of the bulk water band is over 65 cm^−1^ lower (at 3398 cm^−1^). This indicates that the water within a multilayer system has a different structure/environment to water in bulk electrolyte, and that the water in the bulk electrolyte is more hydrogen bonded than water removed from the multilayer^38^. It should be noted that if an anomalous dispersion correction is applied to the removed water spectra, assuming that an evanescent wave is established at the ZnSe/multilayer interface, the peak maximum of the O-H band of the removed water shifts even higher in wavenumber (and further from that of the corrected bulk electrolyte peak maximum).

This observation of water structure in a PSS/PAH polyelectrolyte multilayer correlates with works by Skinner^37^ and Pieniazek^43^, who used FTIR spectroscopy to study water confined within reverse micelles. They found that water within smaller micelles displayed less hydrogen bonding (thus, was shifted to higher wavenumbers) than bulk water. They reported that in spectroscopy, the largest changes in the O-D stretch (which should correlate to changes in the O-H stretch) were found when observing hydrogen bonding between two water molecules, and a water molecule and an anion^44^. In particular, Skinner^37^ and Pieniazek^43^ showed that when hydrogen bonding occurs between water and the sulfonate groups of the surfactant in the micelle the spectra show less hydrogen bonding occurring than in bulk water. The opposite has been found to be true with more/stronger hydrogen bonding between water and phosphate groups in lipid-bilayers^35,45^. Therefore, the sulfonate groups of the PSS within the multilayer system are likely to be the main culprit responsible for the reduced hydrogen bonding seen in the water removed from the film.

## Conclusions

A new soft contact liquid cell with precise control of the sample-crystal separation was developed specifically for studying thin films under confinement in an aqueous environment using synchrotron FTIR microspectroscopy. This technique enables the direct measurement of hydration water within thin films, as well as gathering chemical information about the film itself. Additionally, it provides the capability to compress the films in a controlled manner and observe changes in hydration or film structure. As an example system, a well characterized synthetic, polyelectrolyte multilayer film was used, and films down to 6 bilayers of PSS and PAH could be probed with good signal-to-noise. As the bilayer number increased, it was possible to observe greater signal from the polymers. Furthermore, by analysing the O-H stretching mode, it was clear that the water within the multilayer film had a lower degree of hydrogen bonding than bulk electrolyte, likely due to the hydration shells surrounding the sulfonate groups of PSS. These results illustrate the capability of the new liquid cell, in combination with synchrotron IR radiation, to probe the chemistry and hydration of a thin film both under confinement and within an aqueous environment.

## Methods

### Chemicals

The following reagents were purchased from Sigma-Aldrich (Australia): polyethylenimine (PEI; branched, 25 kDa), poly(sodium 4-styrenesulfonate) (PSS; 70 kDa), poly(allylamine hydrochloride) (PAH; 50 kDa). Potassium chloride (KCl; 99% AR) was purchased from Chem-Supply (Australia). Further purification of KCl was performed in order to remove organic residues by calcination at 550 °C for 8 hrs, followed by recrystallisation and a second recalcination. Cleaning of surfaces was performed using ethanol 100% undenatured (AR grade, Chem-Supply, Australia), Hellmanex (Hellma Analytics, Germany), pH 7 Tickopur R 30 and pH 3 Tickopur TR 3 surfactants (Bandelin, Germany).

### Solution and Sample Preparation

Milli-Q water with resistivity of 18.2 MΩ cm, interfacial tension of 72.4 mN m^−1^ at 22 °C and total organic carbon component of <4 mg L^−1^ was used for the preparation of all solutions. The background electrolyte was 0.15 M KCl solution. Volumetric grade solutions of KOH and HCl (both Merck, KGaA,Darmstadt, Germany) were used to adjust pH of the background electrolyte to 6. PEI (500 ppm), PAH (500 ppm), PSS (500 ppm) solutions were prepared with the 0.15 M KCl, pH 6 solution and stirred overnight. PAH and PSS solutions were then pH adjusted to pH 6 before experiments and used within 24 hrs of preparation. PEI was used at its native pH and used for up to one week after preparation.

Multilayers were formed using the layer-by-layer (LbL) technique^46^. Such films are formed by depositing alternating layers of oppositely charged polyelectrolytes with rinse steps in between. The adsorption step was 15 min followed by a 5 min rinse in 0.15 M KCl, pH 6 solution. PEI was used as anchoring layer, giving a positively charged surface. The sequential adsorption of PSS (a polyanion with a pKa of 1.0^47^) and PAH (a polycation with a pKa of 9.5^47^) was repeated until the requisite number of bilayers were formed; (PEI/(PSS/PAH)_x_, where x is the required number of bilayers.

### ATR-FTIR Spectroscopy

ATR-FTIR experiments were performed using a Varian 670-IR FTIR spectrometer (Agilent Technologies, USA) with a fitted Fast-IR single bounce ATR accessory, using a ZnSe ATR crystal and a liquid flow cell. Solutions were pumped into the cell using Tygon Precision tubing (Masterflex L/S 13, Cole Parmer, USA) and a peristaltic pump (Masterflex L/S, John Morris Scientific, Australia).

Prior to standard ATR-FTIR analysis, the zinc selenide (ZnSe) total internal reflection element (crystal) was polished in a figure-of-eight pattern for 2 min with colloidal silica suspension (OP-U, Struers, Denmark) on a wet, MD-Nap™ 250 mm polishing pad (Struers, Denmark). The polishing pad and crystal were rinsed and the crystal was buffed for an additional minute with Milli-Q water. The crystal was then sonicated for 30 min in 2% pH 7 Tickopur, before rinsing with Milli-Q water and further sonication in 100% undenatured ethanol for 15 min. Finally, the crystal was rinsed, wiped with ethanol, rinsed for the final time, and dried in a nitrogen stream (99.999% purity, BOC, Australia) before mounting in the liquid cell device.

Multilayer films were formed on the ZnSe crystal surface under flow conditions, where polymer and rinsing solutions were introduced into the ATR cell at a flow rate of 0.333 mL.min^−1^ and 1.000 mL.min^−1^, respectively. Experimental data was obtained for three independent experiments. The same representative data set is presented throughout.

Agilent Resolutions Pro software v5.2.0. was used to collect single channel spectra using 256 co-added scans obtained in the spectral region of 4000–650 cm^−1^ with 4 cm^−1^ resolution^21^. Spectra were recorded in the following order: (*i*) a background spectrum of the ZnSe crystal in air; (*ii*) a water vapour (WV) spectrum in air, recorded 15 min after the background; (*iii*) a spectrum of the background electrolyte (KCl); and, (*iv*) polymer spectra recorded after each successive rinse step. The KCl spectrum is used to manually subtract the background electrolyte from the spectra taken after each adsorption step to flatten the O-H bending mode at ~1630 cm^−1^, which resulted in the corrected spectra of multilayer build-up. Spectra of individual layer were produced by subtracting the spectra of the previous layer from the one above in a 1:1 ratio. Spectra of multilayer build-up have also been presented differently by subtracting the KCl spectrum in a 1:1 ratio. Spectra of individual solution were recorded from the bulk solution *in situ*. The WV spectrum was used to remove water vapour peaks present in all the above spectra. OMNIC software v8.2.0.387 (Thermo Fisher Scientific, USA) was used for data processing.

### Synchrotron FTIR Microspectroscopy

For synchrotron FTIR 4.5 × 4.5 mm squares of silicon wafers (p-type, <100>, Si-Mat Silicon Materials, Germany) were used as a solid support for gold film sputtering. The sputtering was performed as previously described^48^. In this study, a 300 nm thick gold layer was used to prevent the appearance of interference fringe on the observed FTIR spectrum. The fringing features had been observed before in our preliminary work using thinner (100 nm thick) gold films.

The gold sputter-coated silicon wafer squares were mounted with epoxy glue (EPOTEK 377, Epoxy Technology, Massachusetts, USA) on magnetic steel disks (6 mm diameter, Ted Pella, USA). These discs were cleaned by soaking in 100% undenatured ethanol for 30 min followed by rinsing twice with Milli-Q water and soaking for 15 min in Milli-Q water. The substrates were then air dried. The ZnSe hemispheres (sample contact surface radius of curvature 20 mm, Crystran, UK) were cleaned by successive rinsing with Milli-Q water and ethanol, before being soaked in ethanol for 5 min, rinsed with Milli-Q water for 5 min, and finally dried under vacuum. In addition, the surface of the piezo-controlled sample stage on which the magnetic disc was mounted inside the liquid cell, was cleaned with ethanol on a cotton bud, then rinsed with Milli-Q water from a clean glass pipette between experiments. PEM sample preparation was *via* a dipping protocol, rather than under flow, this method has shown to give thicker PEMs but otherwise, build-up proceeds in a similar manner^49^.

The synchrotron FTIR experiment was performed at the Infrared Microspectroscopy (IRM) beamline (Australian synchrotron, Victoria, Australia), using a Bruker Vertex 80 v spectrometer coupled with a Hyperion 2000 FTIR microscope and a liquid nitrogen-cooled narrow-band mercury cadmium telluride (MCT) detector (Bruker Optik GmbH, Ettlingen, Germany). All the synchrotron FTIR spectra were recorded within a spectral range of 3900‒750 cm^−1^ using 4 cm^−1^ spectral resolution and 128 co-added scans. Blackman-Harris 3-Term apodization, Mertz phase correction, and zero-filling factor of 2 were set as default acquisition parameters using OPUS 7.2 software suite (Bruker Optik GmbH, Ettlingen, Germany).

Prior to sample measurement, background and water vapor (WV) spectra were collected with a gold substrate mounted on the sample support inside the liquid cell. To perform the background measurement, the piezo-stage was moved to a lowered position, and the gold substrate was placed onto the sample support post, then the ZnSe crystal in the magnetic mount was fitted into the device. Subsequently, the piezo-stage was moved upwards to allow the gold substrate to make a soft contact to the bottom sensing surface of the ZnSe crystal. When the gold substrate was close to making the first contact with the ZnSe crystal, Newton’s rings appeared as a series of concentric circles and could be clearly observed through the microscope. The crystal was determined to be in contact with the sample surface when the Newton’s rings no longer appeared to be increasing in radius upon moving the sample further by 50 nm towards the crystal, but the innermost circle (*i.e*. contact area) became larger in size. The crystal was centered by simultaneously adjusting the positions of the screws in the mount and moving the piezo-stage up and down, to ensure the Newton’s rings were in the center of the visible field of view of the microscope. With the clean crystal in place and the synchrotron-IR beam focused at the centre of the curved underside while in contact with the gold substrate, a background scan was taken (firm contact provides a better background than initial contact). The WV scan was taken 10 min after the background scan. This procedure was performed before every sample.

After that, the PEM sample was placed on the piezo-controlled sample holder. A background electrolyte was introduced onto the sample and the top part of the cell bearing the hemispherical ZnSe crystal was mounted. The piezo-stage was moved up upwards until an initial contact between the ZnSe crystal and a PEM terminated sample was formed (as indicated by the appearance of Newton’s rings). Once the initial contact was formed, the first sample spectra were recorded. The initial contact was determined optically, but confirmed by inspection of the spectral baseline (spectra obtained out of contact had a significantly different baseline due to changed optical properties relative to the background spectrum – obtained from an in-contact spectrum of the gold-ZnSe interface). The multilayer film was then compressed, by translating the piezo-stage in 50 nm increments, towards the ZnSe crystal. The spectra were recorded after each 50 nm step. When the O-H stretching mode showed the same intensity/absorbance after two consecutive compressions then the first of the two was considered to represent the final compression (*i*.*e*. if the 250 nm and 300 nm compressions gave the same intensity of O-H stretching band the 250 nm compression was considered to be the final compression). Note that OPUS software v7.2.139.1294 (Bruker Optics) was used for the spectral data collection, whilst OMNIC software v8.2.0.387 (Thermo Fisher Scientific) was used for manual post-processing manipulation of the spectral data. Two samples of each bilayer number 6, 8 and 10, were measured and are presented in Supplementary Figure S2.

## Electronic supplementary material

supplementary information

## Data Availability

The datasets generated during and/or analysed during the current study are available from the corresponding author on reasonable request.

## References

[1] Wisner, B. In *Comet/Asteroid Impacts and Human Society: An Interdisciplinary Approach* 437–447 (2007).

[2] Shinzawa H, Turner B, Mizukado J, Kazarian SG (2017). Protein hydration in living cells probed by Fourier transform infrared (FT-IR) spectroscopic imaging. Analyst.

[3] Yashunsky V (2010). Infrared Surface Plasmon Spectroscopy of Living Cells. AIP Conference Proceedings.

[4] Golosovsky M, Lirtsman V, Yashunsky V, Davidov D, Aroeti B (2009). Midinfrared surface-plasmon resonance: A novel biophysical tool for studying living cells. Journal of Applied Physics.

[5] Vaccari L (2017). Films of bacteria at interfaces. Advances in Colloid and Interface Science.

[6] Schlenoff JB, Rmaile AH, Bucur CB (2008). Hydration contributions to association in polyelectrolyte multilayers and complexes: Visualizing hydrophobicity. Journal of the American Chemical Society.

[7] Chollet B (2016). Multiscale Surface-Attached Hydrogel Thin Films with Tailored Architecture. ACS Applied Materials & Interfaces.

[8] Kim C-L, Kim D-E (2017). Durability and Self-healing Effects of Hydrogel Coatings with respect to Contact Condition. Scientific Reports.

[9] Lei Z, Wu P (2018). A supramolecular biomimetic skin combining a wide spectrum of mechanical properties and multiple sensory capabilities. Nature Communications.

[10] Kroning A (2015). *In Situ* Infrared Ellipsometry for Protein Adsorption Studies on Ultrathin Smart Polymer Brushes in Aqueous Environment. ACS Applied Materials & Interfaces.

[11] Higaki Y, Kobayashi M, Murakami D, Takahara A (2016). Anti-fouling behavior of polymer brush immobilized surfaces. Polymer Journal.

[12] Higaki Y (2015). Versatile inhibition of marine organism settlement by zwitterionic polymer brushes. Polymer Journal.

[13] Shang C, Liu ZP (2011). Origin and activity of gold nanoparticles as aerobic oxidation catalysts in aqueous solution. Journal of the American Chemical Society.

[14] Zhang Y (2018). Fit-for-purpose block polymer membranes molecularly engineered for water treatment. npj Clean Water.

[15] Zahn R (2014). The entropy of water in swelling PGA/PAH polyelectrolyte multilayers. Soft Matter.

[16] Crouzier T, Picart C (2009). Ion pairing and hydration in polyelectrolyte multilayer films containing polysaccharides. Biomacromolecules.

[17] Aggarwal N (2013). Tuning cell adhesion and growth on biomimetic polyelectrolyte multilayers by variation of pH during layer-by-layer assembly. Macromol. Biosci..

[18] Aggarwal N, Groth T (2014). Multilayer films by blending heparin with semisynthetic cellulose sulfates: Physico-chemical characterization and cell responses. Journal of Biomedical Materials Research - Part A.

[19] Beattie DA (2012). Synchrotron FTIR microscopy of Langmuir-Blodgett monolayers and polyelectrolyte multilayers at the solid-solid interface. Langmuir.

[20] Benbow NL (2017). The influence of polyanion molecular weight on polyelectrolyte multilayers at surfaces: protein adsorption and protein-polysaccharide complexation/stripping on natural polysaccharide films on solid supports. Physical Chemistry Chemical Physics.

[21] Ho TTM (2015). *In Situ* ATR FTIR Spectroscopic Study of the Formation and Hydration of a Fucoidan/Chitosan Polyelectrolyte Multilayer. Langmuir.

[22] Müller M (2006). pH dependence and protein selectivity of poly(ethyleneimine)/poly(acrylic acid) multilayers studied by *in situ* ATR-FTIR spectroscopy. Biomacromolecules.

[23] Heuvingh J, Zappa M, Fery A (2005). Salt Softening of Polyelectrolyte Multilayer Capsules. Langmuir.

[24] Miller MD, Bruening ML (2005). Correlation of the Swelling and Permeability of Polyelectrolyte Multilayer Films. Chemistry of Materials.

[25] Iturri Ramos JJ, Stahl S, Richter RP, Moya SE (2010). Water Content and Buildup of Poly(diallyldimethylammonium chloride)/Poly(sodium 4-styrenesulfonate) and Poly(allylamine hydrochloride)/Poly(sodium 4-styrenesulfonate) Polyelectrolyte Multilayers Studied by an *in Situ* Combination of a Quartz Crystal Microbalance with Dissipation Monitoring and Spectroscopic Ellipsometry. Macromolecules.

[26] Schwarz B, Schönhoff M. (2002). Surface Potential Driven Swelling of Polyelectrolyte Multilayers. Langmuir.

[27] Abbott SB (2014). Hydration of odd-even terminated polyelectrolyte multilayers under mechanical confinement. Macromolecules.

[28] De Vos WM (2013). Nonuniform hydration and odd-even effects in polyelectrolyte multilayers under a confining pressure. Macromolecules.

[29] Dodoo S, Steitz R, Laschewsky A, von Klitzing R (2011). Effect of ionic strength and type of ions on the structure of water swollen polyelectrolyte multilayers. Physical Chemistry Chemical Physics.

[30] Koehler R, Steitz R, Von Klitzing R (2014). About different types of water in swollen polyelectrolyte multilayers. Advances in Colloid and Interface Science.

[31] Schönhoff M (2007). Hydration and internal properties of polyelectrolyte multilayers. Colloids Surf., A.

[32] Ii, K., Ohshio, S., Akasaka, H. & Siaitoh, H. In *IOP Conf. Series: Materials Science and Engineering* Vol. 18 (IOP Publishing, 2011).

[33] Peterson AW, Halter M, Tona A, Plant AL (2014). High resolution surface plasmon resonance imaging for single cells. BMC Cell Biology.

[34] Auer BM, Skinner JL (2008). IR and Raman spectra of liquid water: Theory and interpretation. The Journal of Chemical Physics.

[35] Gruenbaum SM, Skinner JL (2011). Vibrational spectroscopy of water in hydrated lipid multi-bilayers. I. Infrared spectra and ultrafast pump-probe observables. The Journal of Chemical Physics.

[36] Skinner, J. L., Auer, B. M. & Lin, Y. S. In *Advances in Chemical Physics*Vol. 142 (ed S. A. Rice) 59–103 (2008).

[37] Skinner JL, Pieniazek PA, Gruenbaum SM (2012). Vibrational Spectroscopy of Water at Interfaces. Accounts of Chemical Research.

[38] Perakis F (2016). Vibrational Spectroscopy and Dynamics of Water. Chemical Reviews.

[39] Woods DA, Bain CD (2014). Total internal reflection spectroscopy for studying soft matter. Soft Matter.

[40] Geissler PL (2013). Water Interfaces, Solvation, and Spectroscopy. Annual Review of Physical Chemistry.

[41] Walrafen, G. E. In *The* Physics *an*d Phy*sical Chemistry of Water* (ed Felix Franks) 151–214 (Springer New York, 1972).

[42] Hopcroft MA, Nix WD, Kenny TW (2010). What is the Young’s Modulus of Silicon?. Journal of Microelectromechanical Systems.

[43] Pieniazek PA, Lin Y-S, Chowdhary J, Ladanyi BM, Skinner JL (2009). Vibrational Spectroscopy and Dynamics of Water Confined inside Reverse Micelles. The Journal of Physical Chemistry B.

[44] Lin YS, Auer BM, Skinner JL (2009). Water structure, dynamics, and vibrational spectroscopy in sodium bromide solutions. The Journal of Chemical Physics.

[45] Zhao W, Moilanen DE, Fenn EE, Fayer MD (2008). Water at the Surfaces of Aligned Phospholipid Multi-Bilayer Model Membranes Probed with Ultrafast Vibrational Spectroscopy. Journal of the American Chemical Society.

[46] Decher G, Hong JD, Schmitt J (1992). Buildup of ultrathin multilayer films by a self-assembly process: III. Consecutively alternating adsorption of anionic and cationic polyelectrolytes on charged surfaces. Thin Solid Films.

[47] Lewis SR (2011). Reactive nanostructured membranes for water purification. Proceedings of the National Academy of Sciences.

[48] Beattie DA (2015). Spectroscopic study of ionic liquid adsorption from solution onto gold. Physical Chemistry Chemical Physics.

[49] Ho TTM (2015). Tuning polyelectrolyte multilayer structure by exploiting natural variation in fucoidan chemistry. Soft Matter.

[50] Li H, Zheng H, Tong W, Gao C (2017). Non-covalent assembly of poly(allylamine hydrochloride)/triethylamine microcapsules with ionic strength-responsiveness and auto-fluorescence. Journal of Colloid and Interface Science.

[51] Molino Cornejo JJ, Matsuoka E, Daiguji H (2011). Size control of hollow poly-allylamine hydrochloride/poly-sodium styrene sulfonate microcapsules using the bubble template method. Soft Matter.

[52] Świderski G, Kalinowska M, Świsłocka R, Wojtulewski S, Lewandowski W (2013). Spectroscopic (FT-IR, FT-Raman and 1H and 13C NMR) and theoretical in MP2/6-311 ++G(d,p) and B3LYP/6-311 ++G(d,p) levels study of benzenesulfonic acid and alkali metal benzenesulfonates. Spectrochimica Acta Part A: Molecular and Biomolecular Spectroscopy.

[53] Yang JC, Jablonsky MJ, Mays JW (2002). NMR and FT-IR studies of sulfonated styrene-based homopolymers and copolymers. Polymer.

[54] Zundel G (1969). Hydration Structure and Intermolecular Interaction in Polyelectrolytes. Angewandte Chemie International Edition in English.

[55] Coates, J. *Interpretation of Infrared Spectra, A Practical Approach*. (2006).

[56] Stuart, B. H. In *Infrared Spectroscopy: Fundamentals and Applications* 71–93 (John Wiley & Sons, Ltd, 2005).

