# Supplementary material for: A Novel Soft Contact Piezo-Controlled Liquid Cell for Probing Polymer Films under Confinement using Synchrotron FTIR Microspectroscopy
Source: Sci Rep. 2018 Dec 13;8:17804. doi: 10.1038/s41598-018-34673-4 (PMC6292912; doi:10.1038/s41598-018-34673-4)
Supplement: Supplementary file 1 — supplementary information [file 41598_2018_34673_MOESM1_ESM.docx]

Supplementary Information for:

**A Novel Soft Contact Piezo-Controlled Liquid Cell for Probing Polymer Films under Confinement using Synchrotron FTIR Microspectroscopy**

Natalie L. Benbow,^1,2^ Jessie L. Webber,^1,2^ Piotr Pawliszak,^1,2^ Damien A. Sebben,^1^ Tracey T. M. Ho, ^1^ Jitraporn Vongsvivut,^3^ Mark J. Tobin,^3^ Marta Krasowska,^1,2^ and David A. Beattie *^1,2^

^1^ Future Industries Institute, University of South Australia, Mawson Lakes, South Australia 5095, Australia.

^2^ School of Information Technology and Mathematical Sciences, University of South Australia, Mawson Lakes, South Australia 5095, Australia.

^3^ Infrared Microspectroscopy (IRM) Beamline, Australian Synchrotron, Clayton, Victoria 3168, Australia.

* Corresponding Author: Email – [David.Beattie@unisa.edu.au](mailto:David.Beattie@unisa.edu.au)

Streaming Potential Measurements

The spinning disk method (ZetaSpin Model 1.2, ZetaMetrix, USA) was used to measure the streaming potential of a surface[^1^](#_ENREF_1). As the disk spins a radial flow of background electrolyte (0.001 M KCl, at pH of multilayer formation) is created across the sample. The mobile ionic charge of the diffuse zone of the electrical double layer flows out to the edges of the rotating disk creating an ionic current. The ionic current then returns to the center of the disk from the bulk electrolyte creating a potential difference, thus a streaming potential arises between the two reference electrodes. The zeta potential can then be inferred from streaming potential measurements [^2^](#_ENREF_2).

The surfaces of sputter-coated glass discs for streaming potential measurements were soaked in 100 % denatured ethanol for 30 min, rinsed in Milli-Q water and dried in a stream of nitrogen. The cleaned disks were adhered to the mounts with 3M VHB acrylic tape (Digikey, USA). A mounted gold-sputtered glass disk was placed above the electrode in 0.001 M KCl, pH 6 solution (of conductivity 120 μS.cm^-1^) in the measuring cell and the streaming potential was measured. The inferred value of zeta potential of gold was - 42 mV. The build-up of the multilayer was then monitored by the determination of zeta potential from streaming potential measurements.

After each polyelectrolyte adsorption and rinse step, the sample was rinsed briefly in 0.001 M KCl, pH 6 solution to minimise changes in ionic strength and conductivity of the background electrolyte within the measurement cell. It should be noted that the conductivity has previously been seen to increase by approximately 10 μS.cm^-1^ over the course of the experiment. However, this change in conductivity has a negligible effect on the value of the zeta potential for the measured system.

The alternating zeta-potential of the surface of the 10 bilayer PSS / PAH multilayer can be seen in Supplementary Figure S1. The figure shows an average of two experiments where each layer was measured once at pH 6. PEI gave a zeta-potential of ~ 44 mV, and as expected the build-up shows an alternating surface charge. PSS shows a negative charge drifting from ~ - 43 mV at bilayer 1 to ~ - 30 mV at bilayer 10. Meanwhile, PAH shows a positive charge of ~ 41 mV at bilayer 1 that decreases in magnitude to ~ 32 mV at bilayer 10.


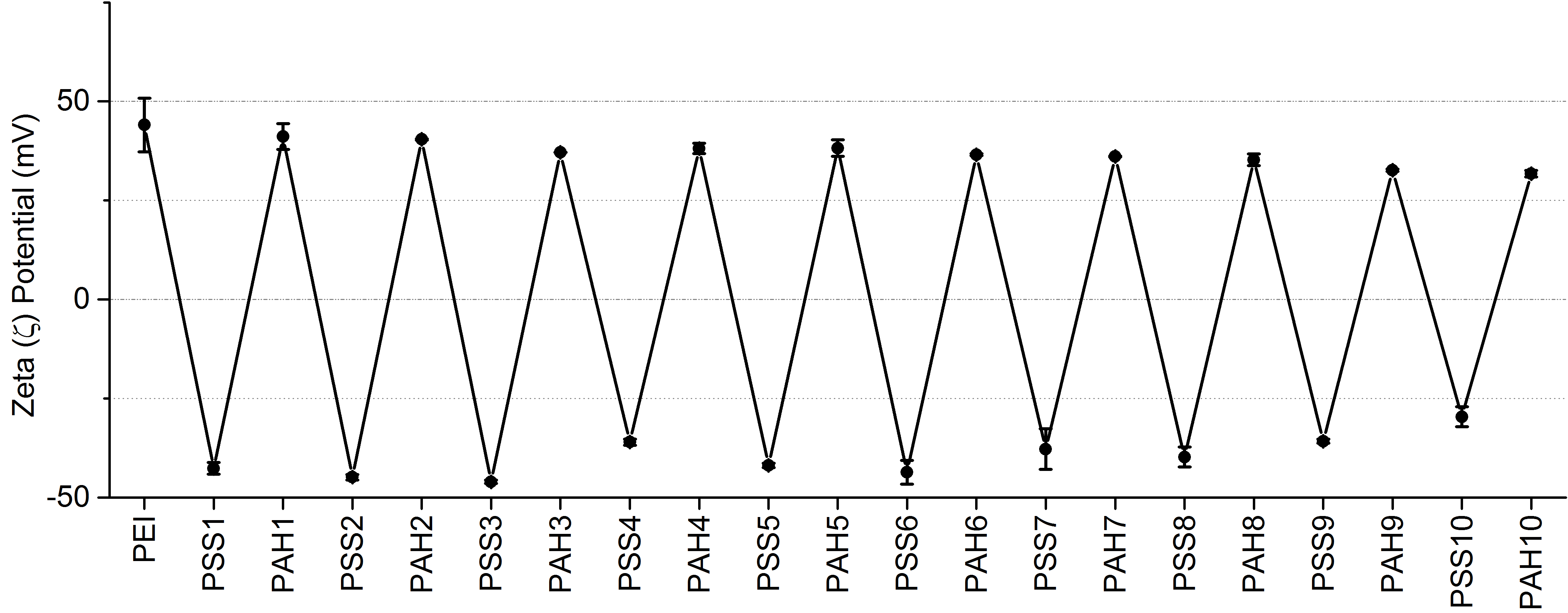


**Supplementary Figure S1**: The zeta-potential at pH 6 for the build-up of a 10 bilayer PSS / PAH multilayer.

The negative surface potential of PSS-terminating multilayers and greater counter ion concentration draws water into the film, making it more hydrated than a PAH-terminating multilayer[^3^](#_ENREF_3)^,^[^4^](#_ENREF_4). In regards to surface charge, Schwarz’s[^5^](#_ENREF_5) NMR results claim the opposite, that positively charged, PAH-terminating layers result in a more hydrated multilayer. Wong[^4^](#_ENREF_4) (who used ellipsometry and contact angle measurements) states that this discrepancy may be due to mobile water within the multilayer that cannot be detected by NMR. However, it may be explained by the fact that Schwarz dialyzed the PSS with a molecular weight cut off of 14,000 kDa before use. So, the molecular weight of the PSS may play a greater role in the hydration of the multilayer than the surface charge. This hydration phenomenon has been shown to be the case with biopolymer polyelectrolyte multilayers[^6^](#_ENREF_6), where low molecular weight polyanions produced a less hydrated multilayer than unfractionated polyanions, as the smaller polyelectrolyte is able to diffuse into the multilayer and displace water.

Elzbieciak’s work showed that PSS / PAH multilayers formed at lower pH (4 – 7) were thicker and had a contact angle of ~20 - 30˚ for PSS and ~45 - 55˚ for PAH at pH 4[^7^](#_ENREF_7). This corresponds to the increased hydrophilicity of PSS over PAH. Additionally, it is known that the potential at the gas / water interface is negative ~ - 35 to - 65 eV [^7^](#_ENREF_7)^,^[^8^](#_ENREF_8) so, the electric double layer potential will be positive, creating a greater attraction between water and the negatively charged PSS layers than the positively charged PAH layers.

Synchrotron FTIR Microscopy

*PEM dipping procedure*: Clean glass petri dishes were used to soak the substrates in the required solution for the allotted time, each sample was moved from one solution to the other using plasma cleaned tweezers. When moving from a polyelectrolyte solution to the KCl rinse, each sample was dipped into two beakers containing KCl to remove any excess polyelectrolyte solution from the substrate and tweezers to minimise the chance for nanoparticles formation. All samples were built using one batch of solutions, and dipped in the same dishes. The solutions were changed every other bilayer to ensure the concentration and purity of the solutions was maintained throughout the experiment.

Spectra were recorded for multilayers composed of 6, 8, and 10 bilayers, and in each case, the polycation (poly(allylamine hydrochloride) was the terminating layer. The samples were measured in duplicate, and spectra were acquired after initial contact, and again after the *z­*- axis piezo drive had been moved 50 nm further into contact. These movements (and spectra acquisition) were repeated until the water band intensity did not change from one compression to the next. The very last compression spectra was discarded (as it was the same as the previous spectra) and the ‘previous’ spectra was used as the ‘final compression’ spectra. These spectra for all three multilayer samples (6, 8, and 10 bilayers) are presented in Supplementary Figure S2.


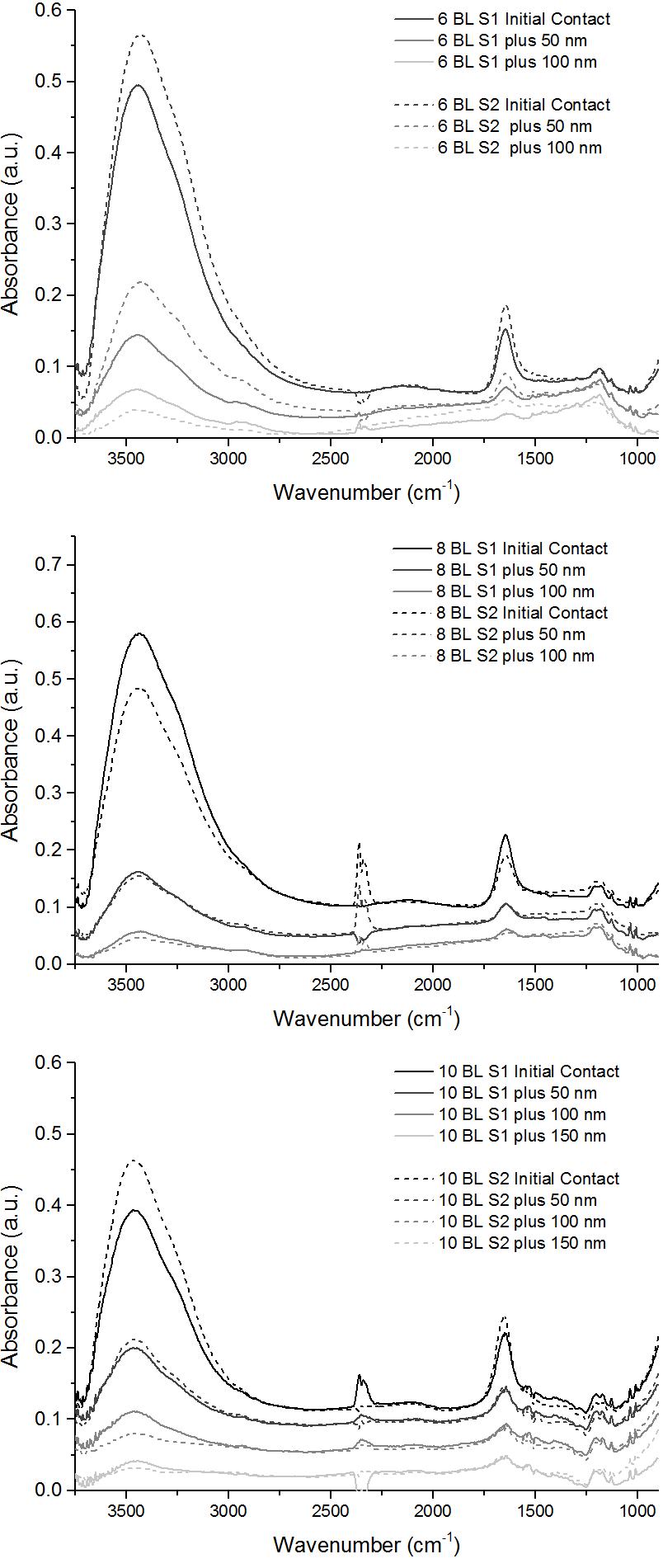


**Supplementary Figure S2**: Synchrotron FTIR microspectroscopy spectra from the solid-solid contact made between a ZnSe hemisphere and a PEM-coated gold substrate, immersed in electrolyte, for different bilayer number multilayers (6, 8, and 10). Spectra are included for two replicates of each multilayer, and for sequential compression, with the piezo drive moving in steps of 50 nm.

**REFERENCES**

1 Sides, P. J., Newman, J., Hoggard, J. D. & Prieve, D. C. Calculation of the Streaming Potential near a Rotating Disk. *Langmuir* **22**, 9765-9769 (2006).

2 Ho, T. T. *et al.* Formation and tribology of fucoidan/chitosan polyelectrolyte multilayers on PDMS substrates. *Biotribology* **12**, 15-23 (2017).

3 Koehler, R., Steitz, R. & Von Klitzing, R. About different types of water in swollen polyelectrolyte multilayers. *Advances in Colloid and Interface Science* **207**, 325-331 (2014).

4 Wong, J. E., Rehfeldt, F., Hänni, P., Tanaka, M. & Klitzing, R. v. Swelling Behavior of Polyelectrolyte Multilayers in Saturated Water Vapor. *Macromolecules* **37**, 7285-7289 (2004).

5 Schwarz, B. & Schönhoff, M. Surface potential driven swelling of polyelectrolyte multilayers. *Langmuir* **18**, 2964-2966 (2002).

6 Benbow, N. L. *et al.* The influence of polyanion molecular weight on polyelectrolyte multilayers at surfaces: protein adsorption and protein-polysaccharide complexation/stripping on natural polysaccharide films on solid supports. *Physical Chemistry Chemical Physics* **19**, 23790-23801 (2017).

7 Elzbieciak, M., Kolasinska, M. & Warszynski, P. Characteristics of polyelectrolyte multilayers: The effect of polyion charge on thickness and wetting properties. *Colloids and Surfaces A: Physicochemical and Engineering Aspects* **321**, 258-261 (2008).

8 Krasowska, M., Kolasinska, M., Warszynski, P. & Malysa, K. Influence of Polyelectrolyte Layers Deposited on Mica Surface on Wetting Film Stability and Bubble Attachment. *The Journal of Physical Chemistry C* **111**, 5743-5749 (2007).
